# Development of an Intra-Layer Adaptive Toolpath Generation Control Procedure in the Laser Metal Wire Deposition Process

**DOI:** 10.3390/ma12030352

**Published:** 2019-01-23

**Authors:** Iker Garmendia, Joseba Pujana, Aitzol Lamikiz, Jon Flores, Mikel Madarieta

**Affiliations:** 1 TEKNIKER, Calle Iñaki Goenaga, 5 20600 Eibar, Spain; joseba.pujana@tekniker.es (J.P.); jon.flores@tekniker.es (J.F.); mikel.madarieta@tekniker.es (M.M.); 2 Department of Mechanical Engineering, University of the Basque Country, Plaza Torres Quevedo 1, 48013 Bilbao, Spain; aitzol.lamikiz@ehu.eus

**Keywords:** laser deposition, metal wire, height control, monitoring, cladding, additive manufacturing, coaxial wire feed, structured light scanning

## Abstract

Recently developed concentric laser metal wire deposition (LMWD) heads allow metal addition processes which are independent of the deposition direction, thus enabling complex paths to be generated. The sensitivity of the process to height deviations has experimentally been observed to be greater with this type of head than with powder ones, therefore requiring more precise and local process control algorithms to be implemented. This work developed a methodology for measuring the part, layer by layer, using a 3D scanner based on structured laser light. Height corrections were applied to the mean and intra-layer height deviations by recalculating the deposition trajectories of the next layer to be deposited. Local height deviations were adjusted by varying the scanning speed, thus increasing the feed rate in the lower areas and decreasing it in the higher ones. Defects generated in the purpose, with height differences within the layer, were successfully corrected. A flat layer was re-established through the application of the control strategy. The internal integrity of the parts due to the scanning speed variation was analyzed, resulting in fully dense parts. The structured light measurement and height correction systems are found to be an affordable and time-efficient solution that can be integrated into an LMWD environment, thereby improving the process robustness.

## 1. Introduction

Laser metal deposition (LMD) is an additive manufacturing (AM) technique in which a high-power laser melts a filler material in the form of powder or wire, resulting in layer-by-layer manufacturing along a predefined robot or machine path [1]. 

AM allows near net-shape components to be produced for specific sectors in a process which is costly. In contrast to subtractive techniques, the amount of raw material required for manufacturing is close to the volume of the final part, requiring only subsequent finish machining to reach the final shape. This characteristic arouses the interest of sectors such as aeronautics or aerospace, in which expensive materials such as titanium and nickel-base alloys are often used. These geometries require either heavy machining operations with high amounts of wasted material [2,3] or costly forging operations prior to machining. The addition of material in wire form means that 100% of the material introduced is melted, resulting in a more efficient, safe, and clean process than in the case of powder LMD. As wire is cheaper than powder, the laser metal wire deposition (LMWD) process is also more cost-efficient [4].

However, there are process robustness barriers that need to be overcome before AM systems are suitable for use in industrial environments. Due to the large number of variables that are handled in the process, it is often difficult to detect the cause of a process failure that may compromise the internal quality of a part and even more so if the failure is the result of a combination of causes. Therefore, new developments in the field of process monitoring and control are still necessary before it is appropriate to incorporate AM technologies into industrial settings [5]. 

Reviews of different LMD monitoring and control methods have been presented [6,7]. Some authors attempt to maintain process stability by employing vision cameras [8,9,10,11,12,13]. By placing a coaxial camera on the deposition head, it is possible to obtain a measurement independent of the deposition direction. In this way, it is possible to monitor the size of the molten pool and control it by varying input parameters such as power or scanning speed. The correct choices for the measurement wavelength range of the camera and filters arranged in the optical path are critical aspects to obtain a suitable measurement. 

LMWD introduces some differences with respect to powder LMD that require a better understanding of the process and new developments regarding in-process control. Some laser welding and repairing heads insert the wire laterally at a defined angle to the processing laser, although this same configuration has also been used to grow parts of simple geometry layer by layer [14]. However, generating geometries with direction changes requires independence in the deposition direction. Wu et al. [15] presented an approach for achieving deposition direction independence in which the welding torch rotated according to the deposition direction in a process of wire arc additive manufacturing (WAAM). Some recently developed LMWD heads [16,17,18,19,20] solve the direction dependence issue by inserting the wire perpendicular to the substrate, so the laser beam is divided within the head and then refocused on the working plane. This introduces the additional complexity of accurately reconstructing the laser beam. Due to the angle of the divided beams with respect to the vertical, the shape of the laser spot changes significantly when varying the working distance. Furthermore, while the defocusing effect is important in powder deposition [21], it is critical in the case of LMWD. Motta and Demir [17] described the issues concerning height deviation using a high-speed vision camera. Defects were observed such as stubbing when the working distance was too small and dripping when it was too large. For this reason, control of the distance between head and part is essential to maintain constant laser power distribution in the working plane and avoid process failures.

By placing a camera off-axis, it is possible to obtain geometric information such as the height of the bead, or the relative position of the robot with respect to the part [22,23,24]. In this way, online corrections of the deposition parameters or the position of the head can be made. However, the cases in which this type of control have been applied are simple geometries, such as single-track walls, and the measurement can give rise to great differences in the case of solid parts with overlapping filling patterns. Donadello et al. [25] developed a novel system of coaxial camera and laser projection which estimated the height of the piece by triangulation. This allowed a measurement of the height during deposition, although the accuracy for more complex geometries was not verified.

In other cases, metrological systems based on triangulation have been investigated. Buhr et al. [26] studied the influence of thermal radiation on the accuracy of the measurements in a line scanner placed on the head and reached the conclusion that, due to the high light emissions of the process, current measurement systems cannot perform accurate measurements during continuous deposition. Interruption of the process makes the precision of the scanner measurement higher than in the case of vision cameras and it is applicable to any geometry. Heralić et al. [27] also introduced a laser line scanner to obtain the height profile of the deposited piece and calculated the wire input for the next layer. 

In this work, a structured light-based scanning technology was employed. A sequence of laser light patterns was projected onto the part and a camera calculated the distance to each point by triangulation. The advantage over a laser line scanning system is that the measurement can be made from an external fixed position without the need to introduce an extra movement, which adds complexity to the system and decreases its accuracy. The developed control strategy complements the work in [28], where a height control strategy based on the recalculation of the deposition trajectories according to the mean height of the previously deposited layer was presented. This paper presents a novel methodology to apply local corrections within the layer, based on the dynamic variation of the scanning speed as a function of the height of each region. The effectiveness of the system was evidenced by the successful correction of induced local defects.

## 2. Materials and Methods 

### 2.1. Description of the laser metal wire deposition (LMWD) Equipment

**Figure 1:**
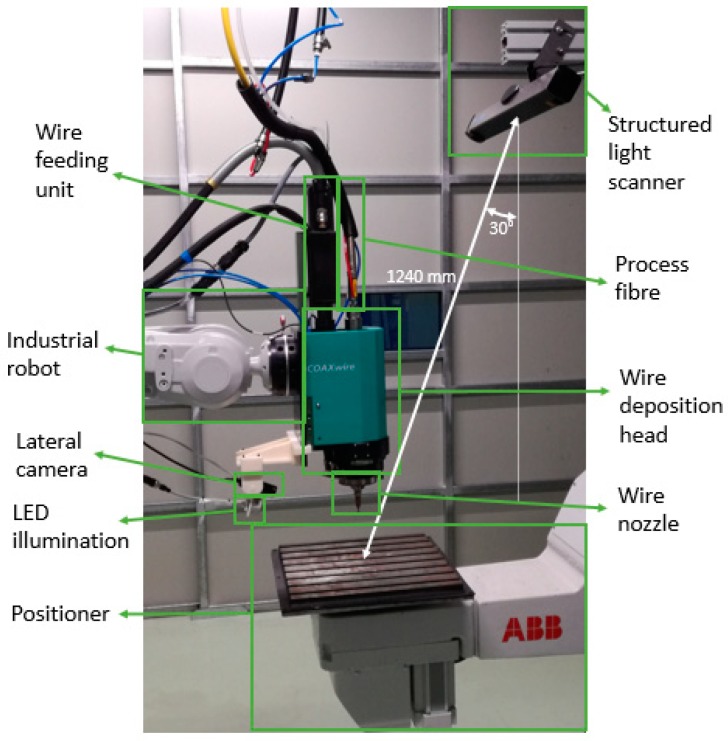
Experimental setup of the laser metal wire deposition (LMWD) robotic cell.

The LMWD cell of Figure 1 was used for the implementation of this work. The processing laser was a 4 kW Ytterbium Laser System YLS4000 (IPG Photonics, Oxford, MA, USA) and the deposition movement was achieved by an IRB4400 robot (ABB, Zurich, Switzerland). 316LSi stainless steel filler material (Lincoln Electric, Cleveland, OH, USA) in the form of 0.8 mm diameter wire was introduced by means of a wire feeder (DINSE G.m.b.H., Hamburg, Germany). The deposition head was a COAXwire (Fraunhofer IWS, Dresden, Germany), which divides the main laser beam into three beams and then focuses them again on the working plane [16]. The result is a direction-independent deposition. The 3D measurement was acquired by means of a Phoxi 3D structured light scanner (Photoneo, Bratislava, Slovakia). The distance between the scanner and the robot positioner was set at 1239 mm as the optimum value recommended by the manufacturer. The point-to-point spacing at this distance was 0.524 mm, considered as adequate for the size of the deviations in the LMWD process. The angle of the scanner with respect to the vertical was fixed at 30° in order to obtain the correct scanning of the upper part of the piece and to avoid collisions with elements of the head.

Prior to deposition, the measuring system must be calibrated to align the scanner coordinate system with the robot’s coordinate system. In this procedure, the points of the target pattern are detected both by vision by the scanner and by touching the points with the robot. With the points referred to in the robot coordinate system, $P_{\mathrm{Robot}}$, and scanner, $P_{\mathrm{Scanner}}$, the transformation matrix T is calculated (Equation (1)). This matrix is applied to all the points of the subsequent scans, also referring the scan points to the robot coordinate system.

(1) $$P_{\mathrm{Robot}}=T\cdot P_{\mathrm{Scanner}}$$

The wire feeding was also monitored continuously by means of a Genie Nano C1940 CMOS vision camera (Teledyne DALSA, Waterloo, ON, Canada) placed laterally and equipped with a visible bandpass filter, a neutral filter, and LED illumination of the work area.

### 2.2. Height Control Methodology

The correction strategy developed calculated the deposition trajectories layer by layer based on the height profile measured in the previously deposited layer. After the deposition of the layer, the process was stopped and the upper surface of the part was scanned. Due to the calibration procedure, the coordinates of the obtained point cloud referred to the same working coordinate system as that of the robot, so height corrections could be applied at their precise location.

In order to reduce the size of the acquired point clouds, in a first step, the points outside the work volume were removed. This reduced point processing time and therefore the stop time between layers.

Based on this scan the trajectories of the next layer were defined. In this case, the procedure of stopping the process, scanning the part, and calculating trajectories was applied for each individual layer. 

**Figure 2:**
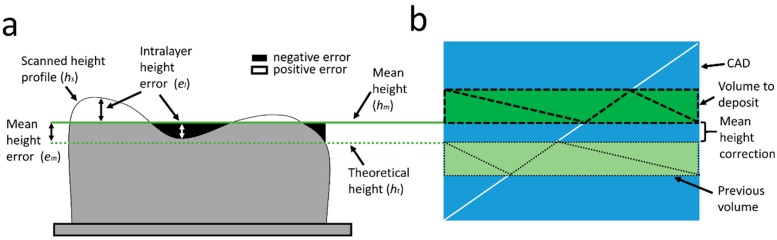
Mean height correction strategy and local deviations. (**a**) Deviations from the mean height of the piece; (**b**) Correction of the mean height by slicing the CAD, in STL format, starting from the mean height of the layer.

A representation of the height deviation of a layer is illustrated in Figure 2a. When a layer is to be deposited, the approximate growth of the layer is known, hence the height that the part should reach after deposition is referred to as theoretical height, $h_{t}$. However, the growth of the part will not necessarily match the theoretical height, so there is an error between the scanned layer height profile, $h_{s}$, and the theoretical height (Equation (2)).

(2) $${e=h}_{s}{-h}_{t}$$

In order to avoid excessive corrections on the part that could lead to faults, the error is divided into a mean, $e_{m}$, and intra-layer, $e_{l}$, height error. The mean error is the difference between the mean height of the scanned layer, $h_{m}$, and the theoretical height, whereas the local error represents the deviation of the scanned height profile with respect to the mean height:

(3) $${e=e}_{m}{+e}_{l}=\left(h_{m}{-h}_{t}\right)+\left(h_{s}{-h}_{m}\right).$$

The control strategy is therefore applied to the height deviation in two steps:

- Mean height correction
- Local intra-layer height correction

Each correction strategy will be explained in the following sections.

#### 2.2.1. Mean Height Correction

The mean height correction based on the robot position adjustment without considering the CAD of the part results in large differences between the theoretical and built geometries, especially in parts with complex shape [29]. Therefore, a control strategy based on the recalculation of the deposition trajectories applied in this work results in a more accurate reproduction of the theoretical geometry (CAD model).

**Figure 3:**
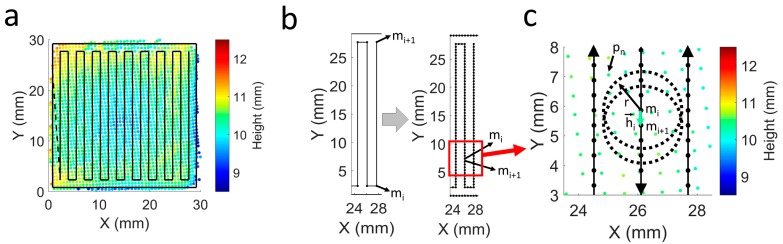
Local height control based on scanned height profile. (**a**) Scanned point cloud and deposition trajectory; (**b**) Discretization step in the trajectory. Left: consecutive points before discretization. Right: consecutive points after discretization; (**c**) Intra-layer local height control strategy.

In order to perform the mean height correction considering the geometry of the part and the scanned data, the same procedure was used as in Reference [28], where the CAD model, in STL format, of the complete part was sliced from the mean measured height to the next height to be reached after deposition of the following layer (Figure 2b). As in this work, only one layer was deposited between scans and the height of the volume to be deposited corresponded to the theoretical layer height. Then, the deposition trajectories of this STL slice were generated using a tool developed in-house [30]. Figure 3a shows the deposition path of a layer together with the scanned point cloud. The deposition pattern consisted of a zig-zag that rotated 90 degrees each layer and one external perimeter.

#### 2.2.2. Local Intra-Layer Height Correction

During experimental work it was found that the mean height correction applied to the whole layer was not sufficient to guarantee the homogeneous and robust growth of the piece. Besides the process parameters commonly referred to, such as power, scanning speed, or wire feed rate, part growth can be affected by other factors such as part heating and cooling rate in different zones, part geometry, or the dynamic precision of the robot in its movements. The importance of each of these factors is often mixed and it is difficult to distinguish their effect on the final result. This paper does not attempt to study the cause of height deviations but rather presents a methodology to control local deviations within the layer regardless of their origin.

The purpose of intra-layer height correction is to introduce more material into areas where the height is insufficient and less material where height is excessive. The principal alternatives for varying the material feed rate in different areas in LMWD are to vary the wire feeding speed or the machine (or robot) scanning speed. In this case, the choice made by the authors was to change the scanning speed, exploiting the ability to modify the deposition trajectories of the subsequent layers to be deposited. As a combined correction of the mean and local height of the layer was applied, the local intra-layer correction was based on deviations with respect to the mean height, giving rise to smoother corrections.

The deposition paths consist of linear point-to-point movements, which constitute the matrix of M coordinates with the consecutive points of the robot movement:

(4) $$M=\left[m_{1}\;m_{2}{\;\ldots \;m}_{i}{\;\ldots \;m}_{I}\right]{,\;m}_{i}=\left\{{m_{i}}_{x}{{\;m}_{i}}_{y}{{\;m}_{i}}_{z}\right\}.$$

To introduce local corrections, the linear movements are discretized by the insertion of intermediate points. The discretization of the trajectory is illustrated in Figure 3b, with the representation of two consecutive points, $m_{i}$ and $m_{i+1}$, before and after the insertion of the intermediate points. The distance between points is calculated according to a value, d, entered by the user. If $\vec{h_{i}}$ is defined as the vector that connects $m_{i}$ and $m_{i+1}$, the number of points to introduce between them, $n_{i}$, is calculated as follows:

(5) $$n_{i}=\mathrm{round}\left(\left|\vec{h_{i}}\right|/d\right)-1,$$

and the coordinates of the intermediate points, $m_{k}$, are then computed:

(6) $$m_{k}{=m}_{i}+\frac{m_{i+1}{-m}_{i}}{n_{i}+1}\cdot {k,\;k=1,\;2,\;\ldots \;,\;n}_{i}.$$

The M matrix is then redefined by inserting the intermediate points. On the other hand, the scanning of the part originates a matrix, P, with the coordinates of the point cloud:

(7) $$P=\left[p_{1}{\;p}_{2}{\;\ldots \;p}_{n}{\;\ldots \;p}_{N}\right],\;p_{n}=\left\{{p_{n}}_{x}{{\;p}_{n}}_{y}{{\;p}_{n}}_{z}\right\}.$$

Figure 3c shows a zoom view of the trajectory points together with the scanned point cloud and the intra-layer height control parameters. With this control methodology, the scanning speed between the newly defined $m_{i}$ and $m_{i+1}$ is determined as a function of the height profile of the scan. 

A certain point in the trajectory, $m_{i}$, is not necessarily coincident with a point in the scan, $p_{n}$, so the height deviation for each trajectory point, ${\mathrm{desv}}_{m_{i}},$ is calculated based on the z component of the scan points that are placed at a distance less than a user-defined value, r:

(8) $${\mathrm{desv}}_{m_{i}}=\mathrm{mean}\left(P\right),\;P=\left\{p_{n,z}|{{(p}_{n,x}{{-m}_{x}}_{i})}^{2}{+(p}_{n,y}{{-m}_{y}}_{i}{)}^{2}{-r}^{2}<0\right\}.$$

**Figure 4:**
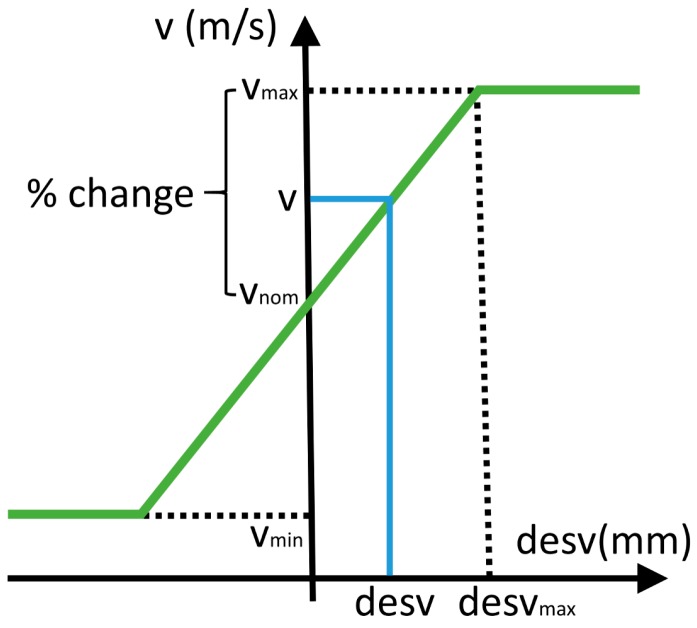
Diagram of scanning speed control based on local height deviation.

The speed of a movement connecting two points, $v_{\vec{h_{i}}}$, is established by means of the diagram shown in Figure 4, taking the mean value of the deviations of the two points being connected:

(9) $$v_{\vec{h_{i}}}{=v}_{\mathrm{nom}}+\frac{v_{\max}{-v}_{\mathrm{nom}}}{{\mathrm{desv}}_{\max}}\cdot \frac{{\mathrm{desv}}_{m_{i}}{+\mathrm{desv}}_{m_{i+1}}}{2},$$

where $v_{\mathrm{nom}}$ is set as the nominal speed from which the scan speed adjustments are made. The slope of the correction curve is also determined by the user-defined parameters ${\mathrm{desv}}_{\max}$ and $v_{\max}$, corresponding to the maximum deviation at which the maximum speed is to be applied. These values determine the severity and stability of the corrections. If the speed change for a certain deviation is excessive, the growth of the part may change more than is necessary to reach the target height, as well as leading to large accelerations during the process. On the other hand, if the speed change entered is insufficient, a local defect may continue increasing and remain uncorrected. 

Finally, speeds higher than $v_{\max}$ and lower than $v_{\min}$ are saturated:

(10) $$v_{\vec{h_{i}}}=\{\begin{array}{c} v_{\min}{,\;\mathrm{if}\;v}_{i}{<v}_{\min}\\ v_{\max}{,\;\mathrm{if}\;v}_{i}{>v}_{\max} \end{array}.$$

The setting of the nominal, maximum, and minimum speeds must also take into account the possible effect of the speed change on the final part quality, in order to guarantee homogeneous component properties and the absence of internal defects.

**Figure 5:**
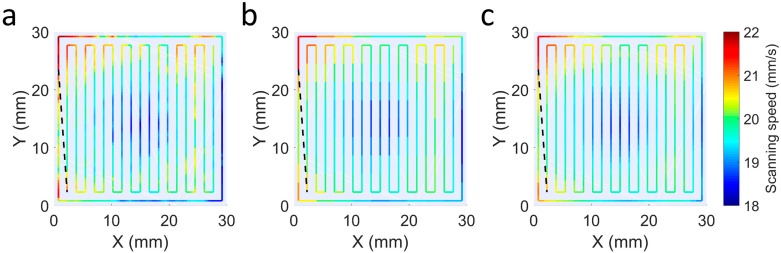
Deposition paths and scanning speeds for different user-entered settings d and r. (**a**) d = 0.5 mm, r = 0.5 mm; (**b**) d = 3 mm, r = 1 mm; (**c**) d = 1 mm, r = 2 mm.

User-defined values d and r also influence performance of the control. Figure 5 shows the deposition trajectories calculated with various combinations of these parameters. In Figure 5a, small r and d values are chosen, resulting in a large number of points on the trajectory and the deviations being calculated with very close scan points. As a result, more localized height variations can be corrected, but the velocity changes are also more pronounced. On the other hand, in Figure 5b, a higher value of d which is also significantly higher than r is selected. This results in fewer points in the trajectories and very discrete and sudden speed changes. Finally, the parameters in Figure 5c were considered as adequate for this work, in which a relatively small value of d and a slightly larger value of r are used. In this way, moderately small deviations can corrected with progressively changing speeds. Although a lower value of d can introduced, the improvement may not be relevant and the calculation time of the trajectories may increase considerably. 

The precision with which the trajectory positions are reached is another aspect to consider. The robot programming allows this precision to be varied in order to allow a continuous movement without speed changes. On the other hand, this means that the real trajectory of the robot does not necessarily correspond with the programmed path. This is the case for the corners of the path in Figure 3a. Although the trajectories are programmed to reach the corner of the section, reaching this point would lead to a significant reduction in the robot’s speed, resulting in excessive growth in the corners. For this reason, the precision of the movement is reduced, resulting in more rounded real trajectories. This is a limitation to consider when determining the point at which corrections are made. In this work, this problem was solved by means of the parameters d and r, which softened this effect to some extent by increasing the distance to the points used to calculate the deviations. In other cases, this aspect can be addressed, for example, by introducing more rounded corners in the trajectory that will enable an accurate movement with the programmed speed.

## 3. Results and Discussion

### 3.1. Influence of Velocity Change on Part Integrity

**Figure 6:**
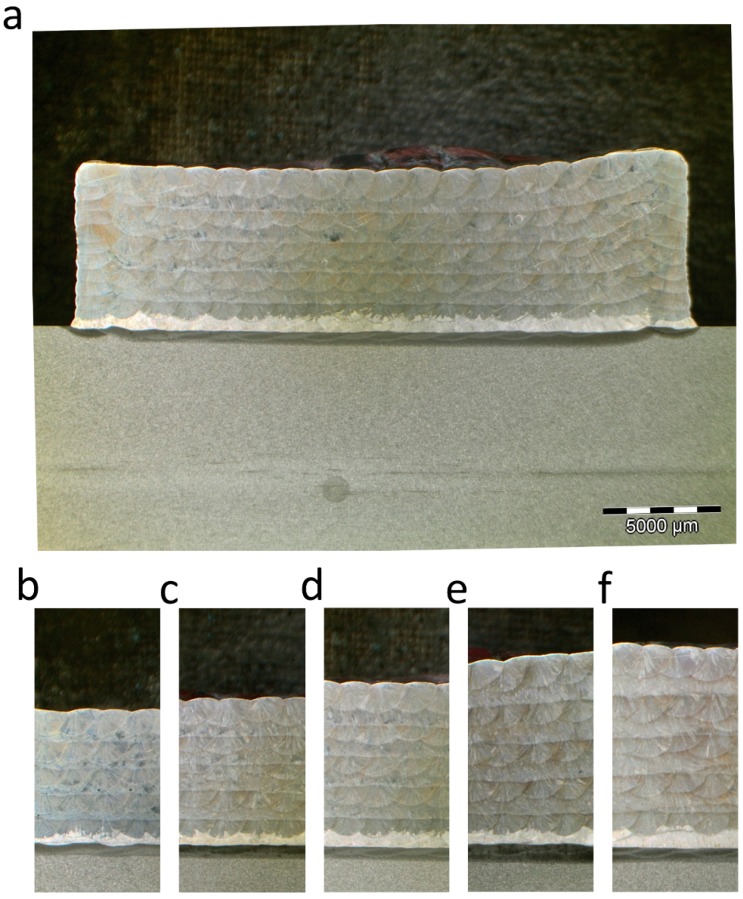
Cross-section of the part for different scanning speeds. (**a**) Complete hexahedron with nominal velocity, 20 mm/s; (**b**) Velocity increase of 20%, 24 mm/s; (**c**) Velocity increase of 10%, 22 mm/s; (**d**) Nominal velocity, 20 mm/s; (**e**) Velocity decrease of 10%, 18 mm/s; (**f**) Velocity decrease of 20%, 16 mm/s.

Initially, 25 mm × 25 mm section hexahedrons with 10 layers were manufactured. The distance between consecutive beads was specified as 1.3 mm. The deposition parameters used are given in Table 1. All parameters were kept constant except the scanning speed of the robot which was varied with respect to the nominal speed, set at 20 mm/min. The height at which each layer was deposited was adjusted based on the mean height of the previous layer. Figure 6a shows the hexahedron manufactured with the nominal speed, whereas in Figure 6a–f hexahedrons varying the speed above and below the nominal speed are presented. Note that as the scanning speed of the robot increased or decreased while maintaining the same deposition trajectories, the height of each layer and the height of the final part increased or decreased accordingly. On the other hand, although the overlap ratio was also changed as a consequence of the bead width change, the hexahedrons obtained showed an absence of pores or internal defects for every velocity.

The results of the experiments have demonstrated the feasibility of adapting the scanning velocity on a part to vary the height of the deposition. Although different scanning speeds affect part heating and cooling rates, thus altering the microstructure of the final manufactured part, this aspect has not been considered in this article, considering as valid totally dense parts, with the absence of pores or cracks. In future research, the effect of these variations on the properties of the final part will be determined.

### 3.2. Defect Correction

Two test specimen geometries were produced to validate the effectiveness of the height correction method. The deposition parameters were again those of Table 1 and the same zig-zag and external perimeter deposition pattern was applied. Although it was proven that parts could be built with a 20% speed change with no defects, the speed in these tests was limited to 10% to soften corrections and ensure internal quality, so the value of $v_{\max}$ was set at 22 mm/s and $v_{\min}$ at 18 mm/s. The maximum height deviation for these limit speeds, ${\mathrm{desv}}_{\max}$, was set at 1 mm.

**Figure 7:**
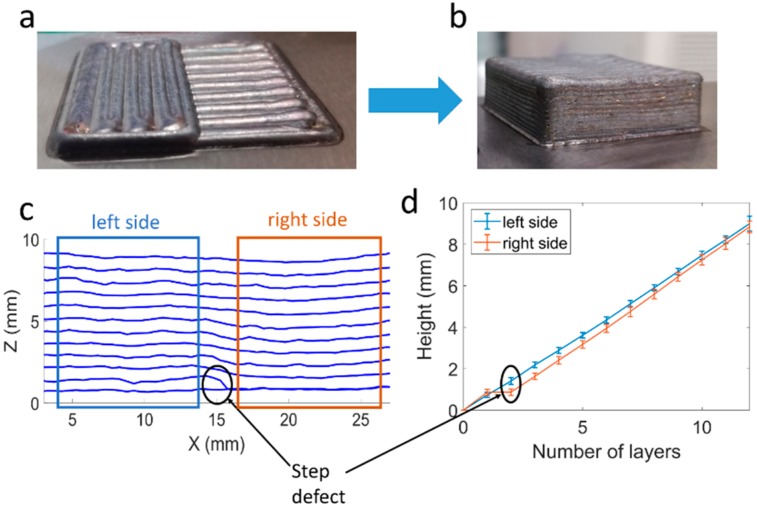
Step defect correction. (**a**) Provoked step defect; (**b**) Part after error correction; (**c**) Height profile for each layer; (**d**) Height evolution for the left and right sides of the part. Mean height and standard deviation.

In the first test (Figure 7a), a step-type defect was simulated. After an initial complete layer deposition, only half of the section was deposited in the second layer. In Figure 7b, the part can be seen after the correction of the step defect, resulting again in a flat surface. Figure 7c shows the height profiles of the part calculated for each layer obtained from the scans, and the provoked step defect. Finally, in Figure 7d, the evolution of the height on both sides of the part is illustrated. The step defect induced in the second layer can be seen, which results in a difference in height between the two sides of the section. This difference in height decreases along the layers until it becomes negligible in the 12th layer.

**Figure 8:**
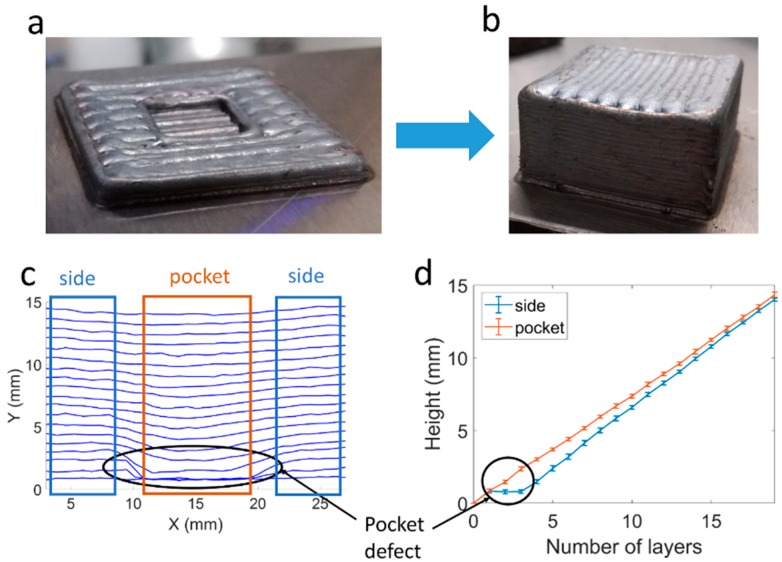
Pocket defect correction. (**a**) Provoked pocket defect; (**b**) Part after error correction; (**c**) Height profile for each layer; (**d**) Height evolution for the central and external zones of the piece. Mean height and standard deviation.

In the second demonstrator (Figure 8a), a pocket-shaped defect was generated. Again, the result of scanning speed control is shown in Figure 8b, in which after a certain number of layers a flat deposition was restored. The pocket defect, consisting of two layers of difference with respect to the top layer height, was produced deliberately (Figure 8c). The evolution of the height for the pocket and for the external zone is reflected in Figure 8d. In this case, the height difference in the third layer was larger, so it was not until the 19th layer that the height of the entire layer was almost levelled.

**Figure 9:**
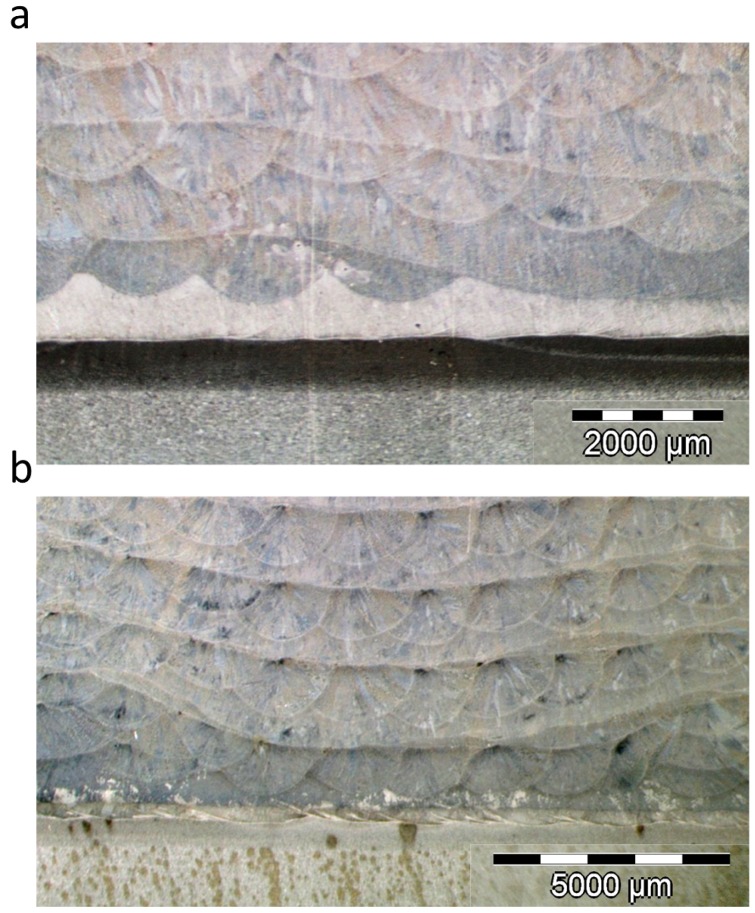
Cross-sections of the samples showing a detailed view of the defect correction. (**a**) Step defect correction; (**b**) Pocket defect correction.

In order to verify that the correction had been applied without compromising the integrity of the part, the corrected samples were cut to analyze possible internal defects. Figure 9 shows a detailed view of the defect correction, in which no interior defect can be seen either in the case of the step defect (Figure 9a) or in the pocket defect (Figure 9b).

By combining the mean layer height correction and local deviations, it was possible to re-establish a constant layer height. While the method presented in this paper was applied to provoked defects, the height correction method developed will be employed in future work on larger parts in order to maintain stability during manufacture. As further points to be developed, the measurement and control strategy will be applied to different geometries and materials, as well as analyzing their effect on the properties of the part.

## 4. Conclusions

A novel in-process height control methodology was developed in the LMWD process based on the measurement of the part by means of a structured light scanner resulting in the correction of intra-layer defects. 

The height correction was implemented in two steps. On the one hand, the average height of the deposited layer was corrected according to the scanned height profile of the workpiece and the reference CAD geometry. On the other hand, the control of the local deviations was based on the variation of the scanning speed of the robot. As a result, it was possible to adapt the deposition rate and therefore the bead height according to the height of each scanned zone.

In order to prove the effectiveness of the height control strategy, defects were provoked to simulate an irregular growth in different areas of the part. The performance of the control was tested, experimentally analyzing how planar layers were re-established after a certain number of deposited layers. 

The methodology proved to be adequate to correct local intra-layer defects. The internal integrity of the part and the absence of defects was also verified in order to validate the process control methodology based on scanning speed variation.

The recalculation of layer-by-layer deposition paths and the combination of mean layer height error corrections and local corrections resulted in a reliable method for introducing the methodology into LMWD manufacturing environments. Although the calibration process, communications, or trajectory programming may be dependent on the manufacturing environment or scanner used, it is considered that the methodology presented is applicable to different machine configurations and measurement systems.

Future research will evaluate the degree to which the system is appropriate for other geometries and materials. 

## Figures and Tables

**Table 1:** LMWD process parameters employed.

Laser Power (W)	Wire Feed Speed (m/min)	Wire Feed Rate (kg/h)	Protective Gas Flow Rate (L/min)	Robot Scanning Speed (mm/s)
1500	3	0.72	12	16–24

## References

[1] Toyserkani E., Khajepour A., Corbin S. (2004). Laser Cladding.

[2] Thomas D., Gilbert S. (2014). Costs and Cost Effectiveness of Additive Manufacturing.

[3] Zhang L.C., Attar H. (2016). Selective laser melting of titanium alloys and titanium matrix composites for biomedical applications: A Review. Adv. Eng. Mater..

[4] Ding D., Pan Z., Cuiuri D., Li H. (2015). Wire-feed additive manufacturing of metal components: Technologies, developments and future interests. Int. J. Adv. Manuf. Technol..

[5] Mani M., Lane B.M., Donmez M.A., Feng S.C., Moylan S.P. (2016). Measurement Science Needs for Real-Time Control of Additive Manufacturing Metal Powder Bed Fusion Processes.

[6] Everton S.K., Hirsch M., Stravroulakis P., Leach R.K., Clare A.T. (2016). Review of in-situ process monitoring and in-situ metrology for metal additive manufacturing. Mater. Des..

[7] Stavropoulos P., Chantzis D., Doukas C., Papacharalampopoulos A., Chryssolouris G. (2013). Monitoring and control of manufacturing processes: A review. Procedia CIRP.

[8] Hofman J.T., Pathiraj B., Van Dijk J., De Lange D.F., Meijer J. (2012). A camera based feedback control strategy for the laser cladding process. J. Mater. Process. Technol..

[9] Hu D., Mei H., Tao G., Kovacevic R. Closed loop control of 3d laser cladding based on infrared sensing. Proceedings of the Solid Freeform Fabrication Symposium.

[10] Ocylok S., Alexeev E., Mann S., Weisheit A., Wissenbach K., Kelbassa I. (2014). Correlations of melt pool geometry and process parameters during laser metal deposition by coaxial process monitoring. Phys. Procedia.

[11] Colodrón P., Fariña J., Rodríguez-Andina J.J., Vidal F., Mato J.L., Montealegre M.Á. FPGA-based measurement of melt pool size in laser cladding systems. Proceedings of the IEEE International Symposium on Industrial Electronics.

[12] Bi G., Gasser A., Wissenbach K., Drenker A., Poprawe R. (2006). Identification and qualification of temperature signal for monitoring and control in laser cladding. Opt. Lasers Eng..

[13] Hu D., Kovacevic R. (2003). Sensing, modeling and control for laser-based additive manufacturing. Int. J. Mach. Tools Manuf..

[14] Baufeld B., Brandl E., Biest O. (2011). Van Der Journal of Materials Processing Technology Wire based additive layer manufacturing: Comparison of microstructure and mechanical properties of Ti–6Al–4V components fabricated by laser-beam deposition and shaped metal deposition. J. Mater. Process. Tech..

[15] Wu Q., Lu J., Liu C., Shi X., Ma Q., Tang S., Fan H., Ma S. (2017). Obtaining uniform deposition with variable wire feeding direction during wire-feed additive manufacturing. Mater. Manuf. Process..

[16] Pajukoski H., Näkki J., Thieme S., Tuominen J., Nowotny S., Vuoristo P. (2016). High performance corrosion resistant coatings by novel coaxial cold- and hot-wire laser cladding methods. J. Laser Appl..

[17] Motta M., Demir A.G. (2018). High-speed imaging and process characterization of coaxial laser metal wire deposition. Addit. Manuf..

[18] Jianjun S., Ping Z., Geyan F., Shihong S. (2018). Geometry characteristics modeling and process optimization in coaxial laser inside wire cladding. Opt. Laser Technol..

[19] Kotar M., Govekar E. (2018). The influence of the workpiece illumination proportion in annular laser beam wire deposition process. Procedia CIRP.

[20] Bambach M., Sizova I., Silze F., Schnick M. (2018). Comparison of laser metal deposition of Inconel 718 from powder, hot and cold wire. Procedia CIRP.

[21] Zhu G., Li D., Zhang A., Pi G., Tang Y. (2012). The influence of laser and powder defocusing characteristics on the surface quality in laser direct metal deposition. Opt. Laser Technol..

[22] Iravani-Tabrizipour M., Toyserkani E. (2007). An image-based feature tracking algorithm for real-time measurement of clad height. Mach. Vis. Appl..

[23] Song L., Bagavath-Singh V., Dutta B., Mazumder J. (2012). Control of melt pool temperature and deposition height during direct metal deposition process. Int. J. Adv. Manuf. Technol..

[24] Davis T.A., Shin Y.C. (2011). Vision-based clad height measurement. Mach. Vis. Appl..

[25] Donadello S., Motta M., Demir A.G., Previtali B. (2018). Monitoring of laser metal deposition height by means of coaxial laser triangulation. Opt. Lasers Eng..

[26] Buhr M., Weber J., Wenzl J.-P., Möller M., Emmelmann C. (2018). Influences of process conditions on stability of sensor controlled robot-based laser metal deposition. Procedia CIRP.

[27] Heralić A., Christiansson A.-K., Lennartson B. (2012). Height control of laser metal-wire deposition based on iterative learning control and 3D scanning. Opt. Lasers Eng..

[28] Garmendia I., Pujana J., Lamikiz A., Flores J., Madarieta M. (2019). Structured light-based height control for Laser Metal Deposition. J. Manuf. Process..

[29] Garmendia I., Leunda J., Pujana J., Lamikiz A. (2018). In-process height control during laser metal deposition based on structured light 3D scanning. Procedia CIRP.

[30] Flores J., Garmendia I., Pujana J. (2018). Toolpath generation for the manufacture of metallic components by means of the laser metal deposition technique. Int. J. Adv. Manuf. Technol..

